# A phase 2, randomized, multicenter, double‐blind, placebo‐controlled trial of S‐adenosyl methionine in participants with mild cognitive impairment or dementia due to Alzheimer's disease

**DOI:** 10.1002/alz.71381

**Published:** 2026-04-14

**Authors:** Sarah Holper, Kevin J. Barnham, Leonid Churilov, Paul Yates, Amy Brodtmann, Sally Johns, Qia‐Xin Li, Oneil Bhalala, Kai Sin Chin, Paula Loveland, Jenny Jia Yu, Colin L. Masters, Rosie Watson, Nawaf Yassi

**Affiliations:** ^1^ Department of Medicine, Melbourne Brain Centre, The Royal Melbourne Hospital University of Melbourne Parkville Victoria Australia; ^2^ Genetics and Gene Regulation, The Walter and Eliza Hall Institute of Medical Research Parkville Victoria Australia; ^3^ National Dementia Diagnostics Laboratory The Florey Institute of Neuroscience and Mental Health Parkville Victoria Australia; ^4^ Department of Medicine Austin Health, University of Melbourne Heidelberg Victoria Australia; ^5^ Medical and Cognitive Research Unit, Department of Geriatric Medicine Austin Health Heidelberg Victoria Australia; ^6^ Department of Neurology Eastern Health, Monash University Box Hill Victoria Australia; ^7^ Department of Geriatric Medicine Modbury Hospital, Northern Adelaide Local Health Network Adelaide South Australia Australia; ^8^ University of Melbourne Parkville Victoria Australia

**Keywords:** Alzheimer's disease, biomarker, clinical trial, S‐adenosyl methionine, tau

## Abstract

**INTRODUCTION:**

S‐adenosyl methionine (SAMe) is a pivotal metabolite in multiple neuronal pathways, including tau dephosphorylation. Reduced SAMe availability has been reported in the Alzheimer's disease (AD) brain, prompting interest in supplementation as a potential therapeutic strategy.

**METHODS:**

This multicenter, randomized, double‐blind, placebo‐controlled phase 2 study recruited people (*n* = 63) with a clinical AD diagnosis. Participants received 180 days of SAMe (400 mg daily) or placebo. Primary outcome was change in plasma phosphorylated tau (p‐tau)217 concentration. Secondary endpoints included safety, tolerability, and cognitive outcomes.

**RESULTS:**

Mean percentage change in plasma p‐tau217 in the SAMe group was an increase of 53.22 (standard deviation [SD] 159.19) compared to 25.34 (SD 94.83) in the placebo group (standardized mean difference 37.58, 95% confidence interval −32.61, 107.76; *p* = 0.288). No significant differences were observed in safety or other secondary endpoints.

**DISCUSSION:**

SAMe did not demonstrate disease‐modifying efficacy at the dose and duration studied; however it was safe and well tolerated.

**TRIAL REGISTRATION:**

ACTRN12620000506998. Registered on the Australian New Zealand Clinical Trials Registry (http://www.anzctr.org.au)

## Highlights

- This study is the first randomized, double‐blind, placebo‐controlled trial of S‐adenosyl methionine (SAMe) in Alzheimer's disease.
- SAMe 400 mg daily for 6 months was safe and well tolerated in participants.
- SAMe did not reduce plasma phosphorylated tau 217 levels compared to placebo.
- There were no significant changes in other biomarkers or cognitive outcomes.
- SAMe monotherapy is unlikely to confer disease‐modifying benefits at this dose and duration.

## 1 BACKGROUND

Plaques containing amyloid beta (Aβ) and neurofibrillary tangles comprising hyperphosphorylated tau are the core pathological hallmarks of Alzheimer's disease (AD), with Aβ toxicity and neurodegeneration being mediated via tau.^1^ Deviations in various molecular pathways drive the evolution of AD pathology including tau hyperphosphorylation, DNA hypomethylation, and neuroinflammation.^2^ S‐adenosyl methionine (SAMe) is a central metabolite in these and many other metabolic pathways on which neuronal homeostasis depends.

AD is associated with significantly reduced concentrations of SAMe in the central nervous system (CNS).^3^, ^4^ Depleting factors may include B12 or folate deficiency, hyperhomocysteinaemia, and excessive SAMe use in maladaptive chronic neuroinflammatory pathways.^5^, ^6^, ^7^ Irrespective of cause, poor CNS SAMe availability would be expected to drive multiple processes underpinning AD pathophysiology.

In murine models of neurodegenerative conditions, SAMe supplementation has been shown to decrease Aβ levels, boost acetylcholine levels, and improve cognition.^8^, ^9^, ^10^ Among humans with AD, SAMe has only been tested as a component of a six‐ingredient nutraceutical formulation, rather than as a stand‐alone supplement. In these studies, supplementation was well tolerated and associated with improvements in cognitive function and dementia symptom severity.^11^, ^12^, ^13^, ^14^

Correcting the SAMe deficiency in the AD CNS via oral supplementation presents an attractive and as yet untested approach to modifying the AD disease process. In this phase 2 trial, we tested the efficacy and safety of this affordable, readily available, oral compound in participants on the AD clinical spectrum.

The primary aim of the trial was to test the hypothesis that treatment with 400 mg per day of oral SAMe for 180 days would lead to a greater reduction from baseline plasma levels of phosphorylated tau (p‐tau)217 compared to placebo in patients with mild cognitive impairment (MCI) or dementia due to AD. Secondary aims were to determine whether, compared to placebo, SAMe treatment would maintain or improve performance as measured by the Repeatable Battery for the Assessment of Neuropsychological Status (RBANS), and be safe.

## 2 METHODS

### 2.1 Overview

**RESEARCH IN CONTEXT**

1. **Systematic review**: The authors conducted a literature search using ClinicalTrials.gov and PubMed for studies of S‐adenosyl methionine (SAMe) supplementation in people with Alzheimer's disease (AD). To our knowledge, this is the first trial to test SAMe as a single‐ingredient intervention in human subjects with AD, rather than as a component of a nutraceutical formulation.
2. **Interpretation**: This phase 2 trial confirmed the tolerability and safety of SAMe at a dose of 400 mg daily for 6 months in people with AD; however, supplementation did not reduce levels of phosphorylated tau (p‐tau)217, p‐tau181, glial fibrillary acidic protein, or neurofilament light chain compared to placebo, nor were there any significant differences in cognitive endpoints.
3. **Future directions**: SAMe did not demonstrate disease‐modifying efficacy at the dose and duration studied; however, it was safe and well tolerated. Future studies could explore alternative dosing strategies, longer treatment periods, or more stable formulations.

This phase 2 randomized, double‐blind, placebo‐controlled trial was prospectively registered on australianclinicaltrials.gov.au (ACTRN12620000506998) and was conducted within the estimand framework for the design and analysis of clinical trials. Briefly, the objective of the estimand framework is to align the clinical trial objectives with the study design, endpoints, and analysis to improve study planning and the interpretation of study results.^15^ The trial design and estimands include prespecified strategies for the conduct of the trial during the COVID‐19 pandemic and is reported according to CONSORT (Consolidated Standards of Reporting Trials) 2025 guidelines.^16^, ^17^, ^18^, ^19^

This study was conducted in accordance with the Declaration of Helsinki and Council for International Organizations of Medical Sciences International Ethical Guidelines. The protocol was approved by Melbourne Health Human Research Ethics Committee. Participants or an appropriate medical treatment decision maker provided written consent to participate in the study and a study partner (individual who has thrice weekly contact with the participant) also provided consent to participate. Participants were recruited across four sites in Australia.

The study protocol has been published previously.^20^ The complete study protocol is available in supporting information (Appendix S1).

### 2.2 Participants and inclusion/exclusion criteria

Community‐dwelling adults aged ≥ 60 years with MCI^21^ or dementia^22^ due to AD according to National Institute on Aging–Alzheimer's Association 2011 criteria were invited to participate.

Eligible participants had a Mini‐Mental State Examination (MMSE) score of at least 18 and had a reliable study partner with whom they had regular interactions to oversee study drug administration and participate in certain study procedures (e.g., the Clinical Dementia Rating [CDR] scale). If taking medications indicated for control of AD symptoms (i.e. cholinesterase inhibitors or memantine), doses must have been stable for at least 8 weeks prior to screening.

Key exclusion criteria included any history of bipolar affective disorder, concurrent use of antidepressant medication, a medical condition other than AD that may contribute to the individual's cognitive impairment, stroke or transient ischemic attack in the past 12 months, clinically significant unstable psychiatric condition in the past 6 months, inability to swallow oral medications, any other medical conditions deemed to limit the individual's life expectancy to < 6 months, participation in another interventional clinical trial, or intake of another investigations drug within 4 weeks or five half‐lives (whichever was longer).

### 2.3 Study visits

Participants completed six study visits. Visit 1 comprised obtaining informed consent, screening for eligibility, enrollment if suitable, and baseline data collection including blood sampling. Randomization and study drug dispensation occurred within the next 28 days (at visit 2). Visits 3 and 4 (at days 30 and 90 post‐randomization, respectively) involved assessments for safety and drug adherence, as well as dispensing additional investigational product. Endpoint data collection occurred at visit 5 (day 180 post‐randomization). Visit 6, 14 days later, comprised a follow‐up telephone call to the participant and study partner for a final adverse event report.

### 2.4 Randomization, blinding, and data management

Participants were randomized within 28 days of enrollment in a 1:1 ratio to receive either 400 mg of SAMe or matched placebo, taken orally once per day for 180 days. Randomization was stratified based on age at enrolment (< 70 years vs. ≥ 70 years) to minimize baseline imbalance between the treatment groups.

A central randomization schedule, generated by an independent statistician who is a part of the data center at the Melbourne Brain Centre at Royal Melbourne Hospital, University of Melbourne, was programmed into an electronic case report form (eCRF) system, housed on a Research Electronic Data Capture (REDCap) platform.^23^, ^24^ The randomization schedule was kept as a secure spreadsheet accessible only to the study manager (employed by the sponsor) and by the data center coordinating the eCRF.

This was a double‐blind study. Treatment allocation was coded to prevent unblinding. Blinded assessors performed all study assessments and remained blinded to treatment allocation until after the database lock at the trial's conclusion.

Data collection and management were facilitated via the REDCap platform.

### 2.5 Investigational product

Study drug and matched placebo were manufactured centrally and provided to participants. The initial investigational drug preparation was a commercially available product containing 400 mg SAMe, which was over‐encapsulated to match an identical placebo preparation. Each participant was allocated three bottles equating to a total of 180 days of treatment (one capsule per day for 180 days).

Approximately halfway through the trial, this preparation became unavailable, and an alternative source of investigational product was developed without unblinding or interruption to study drug administration for any participants. The active study drug and matched placebos were manufactured using commercially available liquid SAMe. Under the second preparation, each bottle contained 120 capsules of SAMe (200 mg soft gel capsules) or matched placebo. Each participant was allocated three bottles equating to a total of 180 days of treatment (two capsules per day for 180 days).

The investigational product was stored in a temperature‐controlled, secure clinical trial drug facility at each study site and dispensed to participants as described.

Participants were instructed to take the assigned treatment once a day for a period of 180 days as an adjunct to their AD standard of care. Adherence was monitored at study visits 2 through 5 inclusively. Participants brought all dispensed bottles to each visit; study staff manually counted any remaining tablets and compared this to the expected quantity to determine adherence.

### 2.6 Assessments and outcomes

#### 2.6.1 Cognitive, functional, and psychological testing

Cognition was evaluated using five instruments: The RBANS,^25^ MMSE,^26^, ^27^ California Verbal Learning Test 3rd edition,^28^ Montreal Cognitive Assessment (MoCA),^29^ and the Digit Symbol Substitution Test.^30^ Functional assessment comprised the CDR scale.^31^ The Geriatric Depression Scale^32^ was also administered.

#### 2.6.2 Biomarker analysis

All blood samples were collected in the morning after an overnight fast. To assess the effect of SAMe on levels of p‐tau217, p‐tau181, neurofilament light chain (NfL), and glial fibrillary acidic protein (GFAP), blood samples (≈ 10 mL per sample) were collected, subaliquoted, and stored at −80°C. Biomarker levels were measured on one of the aliquots using Elecsys in vitro quantitative immunoassays (Elecsys p‐tau217 plasma assay, Elecsys p‐tau181 plasma assay, Elecsys NfL plasma assay, and Elecsys GFAP plasma assay; Roche Diagnostics Australia) on the automated Roche Cobas e402 system at the national dementia diagnostics laboratory. The operator was blinded to the sample status.

Patient plasma was thawed overnight at 4°C, thoroughly mixed by low‐speed vortexing, and any remaining solid materials separated by centrifugation at 10,000 g for 5 minutes. The assay was performed strictly following the manufacturer's protocol. All four biomarkers for each participant, including baseline and follow‐up samples, were measured at the same time using the same aliquot in a single assay run with the same batch of reagents to minimize inter‐assay variability.

#### 2.6.3 Other sample analyses

Apolipoprotein E (*APOE*) genotyping was performed using a custom iPLEX Gold assay on the MassARRAY platform (Agena Bioscience) targeting the two single nucleotide polymorphisms (rs429358 and rs7412) that define *APOE* alleles, allowing classification of participants as *APOE* ε4 carriers or non‐carriers.

Homocysteine levels were determined from fasting blood samples kept on ice until the time of analysis. Levels of ≥ 12 umol/L were considered elevated, as per the reporting laboratory's reference range.

### 2.7 Statistical analyses

Given that establishing biological efficacy was the primary objective of the trial and adhering to the estimand framework principles, the population included in the primary efficacy analysis comprised all participants who achieved at least 80% study drug adherence and where no intercurrent event prevented collection of the primary outcome (i.e., a 180‐day blood sample). This constitutes a principal stratum approach to the management of intercurrent events within the estimand framework and was prospectively prespecified in the protocol and Statistical Analysis Plan.

The primary efficacy outcome was the percentage change in plasma p‐tau217 concentration between baseline and 180 days, with the population‐level outcome measure being mean percentage change in p‐tau217. This outcome was tested using an ANCOVA (analysis of covariance) model with treatment arm as an independent variable and baseline p‐tau217 concentration as a covariate. Standardized mean differences in percentage change with respective 95% confidence intervals (CIs) were reported using a G computation approach.

Secondary estimands (all changes refer to those between baseline and endpoint) included:

‐ The total scaled RBANS score change (population‐level outcome measure: mean percentage change in RBANS score)
‐ Percentage changes in the concentrations of plasma p‐tau181, GFAP, NfL (population‐level outcome measure: mean percentage change in each of these biomarkers).

Secondary efficacy estimands were tested using similar ANVOCA models to the primary estimand with appropriate adjustment for baseline values.

For the primary safety estimand the population was expanded to include all participants who had taken at least one dose of study medication. The population‐level outcome measure for this estimand was the proportion of participants with one or more serious adverse event.

For all primary and secondary estimands, we also performed (as supplementary estimands) analyses including all participants who provided a blood sample, regardless of drug adherence.

Prespecified subgroup analyses were performed to complement evidence from the primary analysis to help to fully characterize the treatment effect.

Analyses were conducted using Stata statistical software version 19.0 (StataCorp). The Statistical Analysis Plan was finalized before database lock and unblinding, and is available in Appendix S2 in supporting information.

### 2.8 Sample size

The total sample size for the study was 60 (30 per arm). Sample size estimation assumed a two‐tailed alpha of 0.05 and a similar baseline standard deviation (SD) between the two arms. A total sample size of 52 participants (26 per arm, allowing for attrition of 4 participants per arm) was estimated to yield 80% power to observe a large treatment effect (Cohen *d* ≥ 0.8). As there was no prior literature to guide an effect size estimation, and given resource and funding limitations at the time of the trial design, this prospective sample size estimation approach was deemed most appropriate.

### 2.9 Safety

An independent data and safety monitoring board (DSMB) oversaw the trial. Interim safety analyses were performed after final data collection from the first 20 participants, and again after 40 participants. The endpoint for this analysis was the proportion of participants with serious adverse events (SAEs) in each arm of the trial. The DSMB—the only people with access to these unblinded interim data—reviewed these results in accordance with the DSMB Charter (Version 1, February 16, 2021). The Haybittle–Peto procedure for generating early stopping boundaries was used^33^; as the Haybittle–Peto boundary (*p* = 0.001, *Z* = 3) at these interim analysis points was not crossed, the independent DSMB recommended continuation of the trial without modification.

## 3 RESULTS

### 3.1 Participants

**FIGURE 1:**
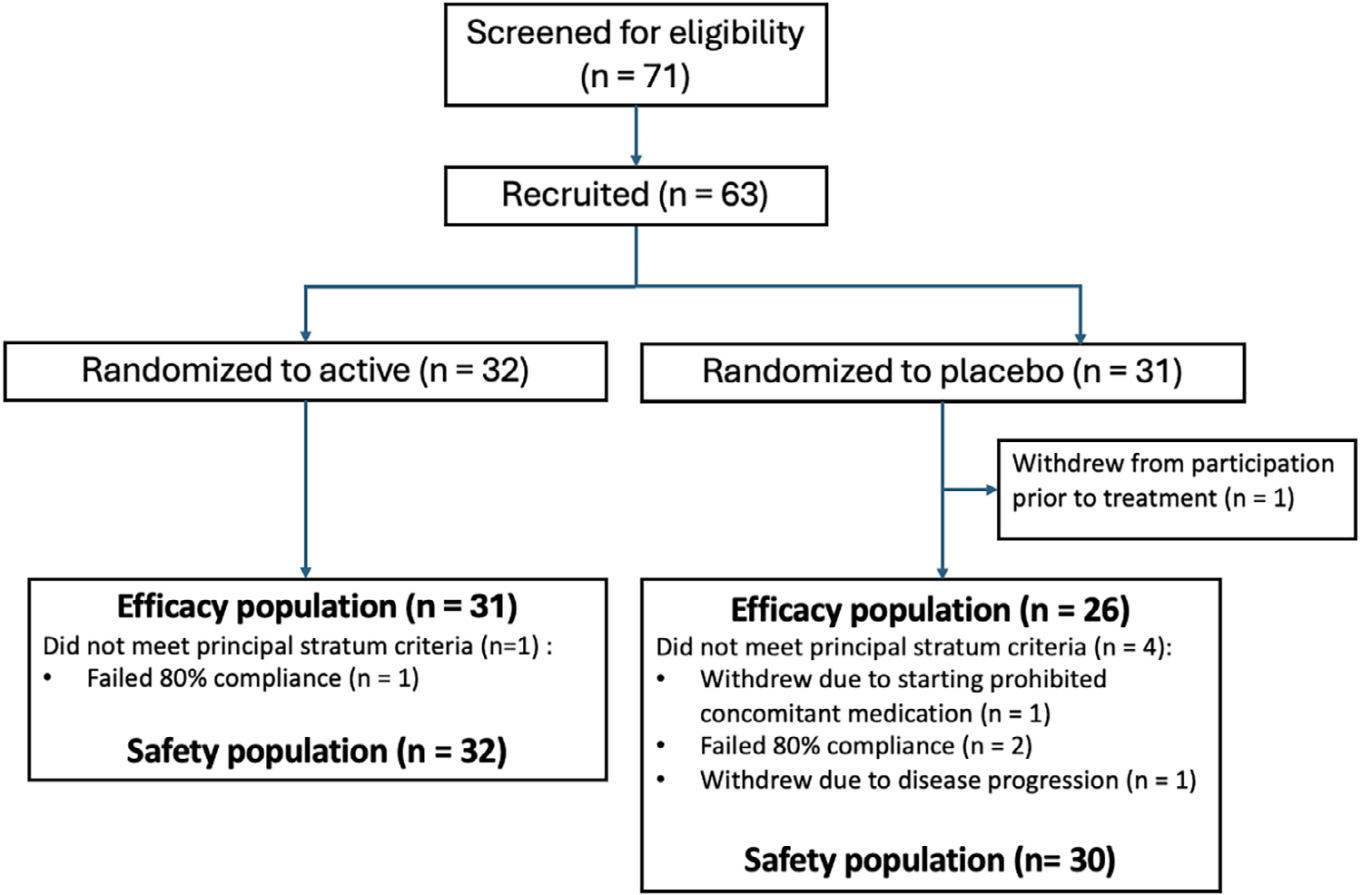
Consolidated Standards of Reporting Trials diagram.

Participants were recruited from four sites between April 2021 and June 2024. Of the 71 patients screened for eligibility, 63 met inclusion criteria and underwent randomization (baseline characteristics of randomized participants are presented in Table S1 in supporting information). Trial completion with provision of a 180‐day blood sample was achieved by 59. Of these, 57 (*n* = 31 active, *n* = 26 placebo) also met the 80% study drug adherence criterion for inclusion in the principal stratum population (Figure 1; Table 1). No major differences in baseline clinicodemographic characteristics were identified between those who did and did not fall within the principal stratum analysis (see Table S2 in supporting information for comparison).

**TABLE 1:** Baseline characteristics of principal stratum population.

	SAMe (*n* = 31)	Placebo (*n* = 26)
Age, years (median, IQR)	76 (70, 81)	77 (72, 80)
Female sex (*n*, %)	16 (51.61)	13 (50)
Years of education (median, IQR)	15 (11, 15)	15 (12, 16)
Presence of ≥ 1 cardiovascular disease or risk factor(s)^a^ (*n*, %)	23 (74.19)	19 (73.08)
AD diagnosis (MCI vs. dementia) (*n*, %)		
AD dementia	16 (51.61)	14 (53.85)
MCI	15 (48.39)	12 (46.15)
Use of AD medications^b^ (*n*, %)	14 (45.16)	11 (42.31)
MMSE (median, IQR)	24 (23, 25)	24.5 (22, 28)
MoCA (median, IQR)	19 (17, 22)	20 (18, 22)
RBANS (mean, SD)	65.68 (12.17)	72.46 (15.38)
GDS‐15 (median, IQR)	2 (1, 4)	2 (1, 3)
CDR global (median, IQR)	0.5 (0.5, 1)	0.5 (0.5, 1)
Baseline p‐tau217, pg/mL (mean, SD)	0.79 (0.54)	0.69 (0.32)
*APOE* ε4 status		
Heterozygous (*n*, %)	21 (67.74)	16 (61.54)
Homozygous (*n*, %)	2 (6.45)	3 (11.54)
Non‐*APOE* ε4 carriers (*n*, %)	8 (25.79)	7 (26.92)

Abbreviation: AD, Alzheimer's disease; *APOE*, apolipoprotein E; CDR, Clinical Dementia Rating; GDS, Geriatric Depression Scale; IQR, interquartile range; MCI, mild cognitive impairment; MMSE, Mini‐Mental State Examination; MoCA, Montreal Cognitive Assessment; p‐tau, phosphorylated tau; RBANS, Repeatable Battery for the Assessment of Neuropsychological Status; SAMe, S‐adenosyl methionine; SD, standard deviation.

^a^ Any of hypertension, dyslipidemia, diabetes, ischemic heart disease, peripheral vascular disease, stroke, transient ischemic attack, atrial fibrillation.

^b^Cholinesterase inhibiters or memantine.

Within the principal stratum population, participants (*n* = 57; female *n* = 29, 50.88%; male *n* = 28, 49.12%) had a median age of 77 years (interquartile range [IQR] 72, 80). Balanced representation was achieved between groups of participants with MCI‐AD (*n* = 27, 47.37%) and dementia due to AD (*n* = 30, 52.63%), with about half being on AD medications (*n* = 25, 43.86%). Median global CDR score was 0.5 (IQR 0.5, 1.0). Most participants were *APOE* ε4 heterozygotes (*n* = 37, 64.91%); a minority were *APOE* ε4 homozygotes (*n* = 5, 8.77%), with the remainder being non‐carriers (*n* = 15, 26.32%). The majority of participants had cardiovascular disease or risk factors (*n* = 42, 73.68%). At baseline, the median MMSE score was 24 (IQR 22, 27) and MoCA score was 19 (IQR 17, 22). Mean baseline RBANS score was 68.77 (SD 14.02).

### 3.2 Efficacy outcomes

**FIGURE 2:**
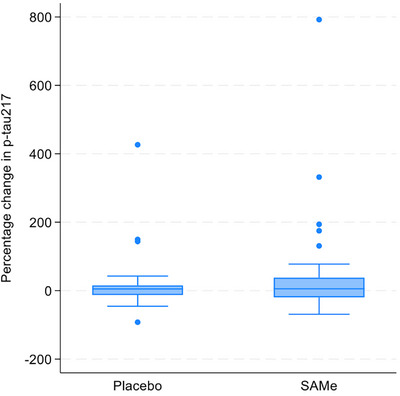
Box plots summarizing per cent change in p‐tau217 from baseline to 180 days for both intervention and placebo groups (principal stratum). Box plots show the median (line within box), IQR (box upper and lower margins), whiskers (range within 1.5 × IQR), and outliers (dots). Negative percent means reduction in p‐tau217 concentration. IQR, interquartile range; p‐tau, phosphorylated tau; SAMe, S‐adenosyl methionine.

**FIGURE 3:**
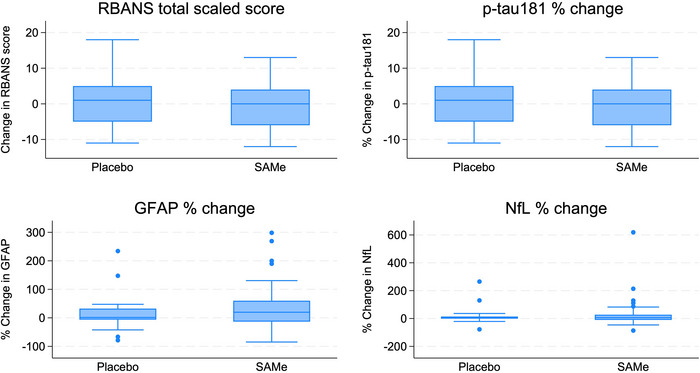
Box plots displaying changes in secondary endpoints (principal stratum). Box plots show the median (line within box), IQR (box upper and lower margins), whiskers (range within 1.5 × IQR), and outliers (dots). Negative percent means reduction in biomarker concentration. GFAP, glial fibrillary acidic protein; IQR, interquartile range; NfL, neurofilament light chain; p‐tau, phosphorylated tau; RBANS, Repeatable Battery for the Assessment of Neuropsychological Status; SAMe, S‐adenosyl methionine.

Mean percentage change in plasma p‐tau217 over 180 days in the SAMe group was 53.22 (SD 159.19) compared to 25.34 (94.83) in the placebo group (standardized mean difference 37.58, 95% CI −32.61, 107.76; *p* = 0.288; Table 2; Figure 2; fold‐change in plasma p‐tau217 presented in Table S3 in supporting information). Mean change in RBANS total scaled score over 180 days was −0.16 (SD 6.11) in the SAMe group compared to 1 (7.74) in the placebo group (standardized mean difference −1.44, 95% CI −5.49, 2.60; *p* = 0.478). No significant differences were observed in other secondary efficacy measures (Table 2; Figure 3).

**TABLE 2:** Study outcomes.

Primary efficacy outcome				
	SAMe (*n *= 31) (mean, SD)	Placebo (*n* = 26) (mean, SD)	Effect size [95% CI]	*p* value
Endpoint p‐tau217, pg/mL	0.94 (0.60)	0.77 (0.51)		
Mean % change in plasma p‐tau217 concentration	53.22 (159.19)	25.34 (94.83)	Standardized mean diff in % change: 37.58 [−32.61, 107.76]	0.288

Secondary outcomes				
	SAMe (*n *= 31) (mean, SD)	Placebo (*n* = 26) (mean, SD)	Effect size [95% CI]	
Mean change in RBANS total scaled score	−0.16 (6.11)	1 (7.74)	Standardized mean diff: −1.44 [−5.49, 2.60]	
Mean % change in p‐tau181concentration	68.10 (183.55)	27.60 (111.32)	Standardized mean diff in % change: 37.15 [−38.08, 112.39]	
Mean % change in GFAP concentration	45.17 (90.37)	14.27 (61.18)	Standardized mean diff in % change: 16.99 [−18.25, 52.22]	
Mean % change in NfL concentration	38.58 (121.26)	16.50 (60.19)	Standardized mean diff in % change: 20.33 [−27.14, 67.81]	

Safety outcome				
	SAMe (*n* = 32)	Placebo (*n* = 30)	Risk difference [95% CI]	p value
Proportion of participants with SAE, *n* (%)	2 (6.25)	1 (3.33)	0.03 [−0.08, 0.13]	0.59

Abbreviation: CI, confidence interval; GFAP, glial fibrillary acidic protein; NfL, neurofilament light chain; p‐tau, phosphorylated tau; RBANS, Repeatable Battery for the Assessment of Neuropsychological Status; SAE, serious adverse event; SAMe, S‐adenosyl methionine; SD, standard deviation.

**FIGURE 4:**
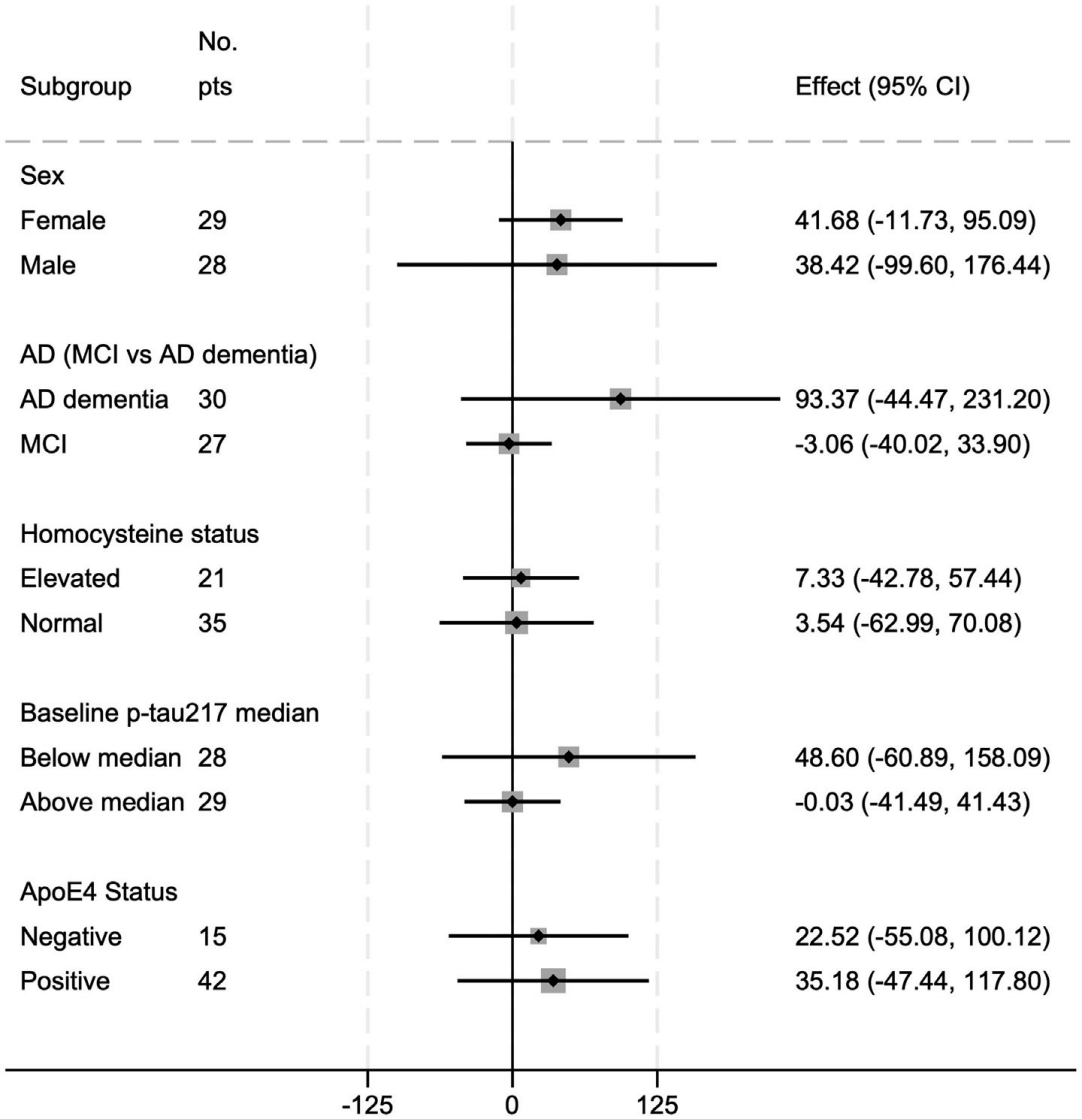
Subgroup analysis of mean difference in percent change in p‐tau217. AD, Alzheimer's disease; *APOE*, apolipoprotein E; CI, confidence interval; MCI, mild cognitive impairment; p‐tau, phosphorylated tau.

Subgroup analysis did not identify a significant effect of SAMe in any prespecified subgroup, nor was there any significant treatment by subgroup interaction observed (Figure 4). In a post hoc analysis stratified by formulation type (over‐encapsulated tablets, liquid soft gels, or a combination), there was no evidence that the effect of SAMe on plasma p‐tau217 differed between formulations (Table S4 in supporting information).

Results for all randomized participants who provided endpoint blood samples (regardless of drug adherence; *n* = 60) are displayed in Figures S1 and S2 in supporting information.

### 3.3 Safety outcomes

Two participants receiving SAMe experienced an SAE during the trial, compared to 1 participant receiving placebo (risk difference 0.03, 95% CI −0.08, 0.13; *p* = 0.59). One participant in the SAMe group experienced a transient ischemic attack, and another experienced symptomatic myocardial ischemia during an outpatient stress echocardiogram. The SAE in the placebo group was ascending cholangitis. All SAEs were deemed definitely not related to the study drug (Table S5 in supporting information).

## 4 DISCUSSION

In this phase 2, randomized, double‐blind, placebo‐controlled trial among 63 people with MCI or dementia due to clinically diagnosed AD, oral SAMe supplementation (400 mg once daily for 180 days) was safe and well tolerated. SAMe supplementation did not reduce p‐tau217, p‐tau181, GFAP or NfL levels compared to placebo, nor were there any significant differences in RBANS scores or other cognitive endpoints observed. These findings indicate that SAMe, at this dose and duration, did not exert measurable disease‐modifying effects in this population.

Several explanations may account for these null findings. Most prominently, SAMe may have no meaningful effect on AD pathophysiology in humans. Additional considerations include the study's modest sample size and short duration of supplementation, potential diagnostic heterogeneity arising from broad inclusion criteria, and pharmacokinetic considerations. Prior studies in AD evaluated SAMe as part of multi‐ingredient nutraceutical formulations, which reported positive effects on cognitive function and dementia symptom severity.^11^, ^12^, ^13^, ^14^ In contrast, our study of SAMe monotherapy did not observe any cognitive benefit or changes in AD‐related plasma biomarkers, suggesting that SAMe alone is unlikely to be responsible for the positive outcomes seen in combination studies.

Our phase 2 trial provides important safety data. SAMe was well tolerated, with no SAEs deemed related to the study drug, no clinically significant safety signals identified, and no participants discontinuing treatment due to intolerance. These findings align with prior evidence indicating that oral SAMe is generally safe across a range of doses.^34^ Emphasizing tolerability is particularly relevant given the older age and frailty of the study population, who may be at increased risk of adverse drug reactions.

An important methodological limitation relates to the trial's pragmatic enrolment criteria, which were intentionally designed to reflect real‐world clinical practice. Trial enrolment required a diagnosis of AD based on clinical consensus criteria. While a biomarker‐supported diagnosis (i.e., a confirmed positive Aβ status) offers the clinician greater certainty regarding underlying AD pathology, the vast majority of patients in our clinical practice receive an AD diagnosis based on clinical criteria alone. Rather than limit our trial's population to the minority of AD patients who have undergone biomarker testing, we took a pragmatic approach to base inclusion on clinical criteria, in keeping with widespread current clinical practice. The small number of participants in this phase 2 study remains another important limitation. Given the absence of robust data to guide a sample size estimation, and limitations in resources and funding, we prospectively powered the trial to observe a large effect size. While the outcome was statistically neutral, our trial nonetheless contributes an important estimate of the effect of SAMe treatment on p‐tau217, and argues against the routine use of SAMe in AD.

The SAMe molecule demonstrates inherent molecular instability and complex pharmacokinetics. It exists in two chiral forms—(S,S) and (R,S)—with only the former diastereomer being biologically active.^35^ When isolated for pharmaceutical preparations, the (S,S) form spontaneously converts to the inactive (R,S) diastereomer, which is unable to be metabolized enzymatically. Due to inevitable degradation, pharmaceutical SAMe preparations typically contain 20% to 30% of the inactive (R,S) diastereomer.^36^ Although strict handling and storage conditions were enforced at study sites, accelerated degradation in participants’ home environments due to elevated temperatures and humidity could not be fully controlled for, potentially reducing drug potency.

Considerable variability exists in the formulation and dosing of oral SAMe used in both clinical practice and research. Oral SAMe is distributed by more than a dozen international manufacturers as an over‐the‐counter supplement in both enteric‐coated and non‐coated forms. Recommended daily doses span 200 to 1600 mg as one to eight tablets taken between one and six times daily. Similar variation occurs in the scientific literature. For example, the aforementioned studies involving SAMe as part of a nutraceutical formulation administered 800 mg daily,^11^, ^12^, ^13^, ^14^ while a 1990 pilot study of oral SAMe in four AD patients used a 1200 mg daily dose.^3^ A systematic review of oral SAMe in non‐dementia CNS disorders (e.g., depression, anxiety, and schizophrenia), reported good tolerability at daily doses between 200 mg and 3200 mg, with mild gastrointestinal complaints being the most common adverse effect.^34^ Importantly, however, this review did not include people with dementia, whose increased age and frailty may heighten their vulnerability to adverse drug reactions.

In the absence of robust data to guide dosing in AD, we prioritized safety by selecting the most commonly advised over‐the‐counter regimen of 400 mg daily. This dose may have been insufficient to exert a biological effect. SAMe's oral bioavailability is low, with absorption occurring primarily in the small intestine.^37^ While the initial over‐encapsulated preparation was enteric‐coated, the subsequent preparation was not, which may have limited systemic absorption. Finally, supplementation for 180 days may be insufficient for SAMe's effects to manifest as measurable biomarker or cognitive changes. Future trials should consider novel adaptive designs to determine the optimal dosing and duration of SAMe supplementation.

While our trial was designed to test whether SAMe supplementation could reduce p‐tau217 compared to placebo, future studies might more pragmatically evaluate whether SAMe helps maintain stable p‐tau217 levels over time rather than achieving outright reductions.

Despite not reaching statistical significance, the point estimates are suggestive of greater increases in mean blood biomarker levels among participants receiving SAMe compared to placebo. There are putative mechanisms by which SAMe supplementation could theoretically exert deleterious metabolic effects, including via its catabolism to toxic methylation inhibitors,^38^ or by disrupting the methionine cycle to promote excess *S*‐adenosyl homocysteine and homocysteine production.^39^ We plan to investigate these considerations using metabolomic and proteomic techniques to better characterize the pharmacokinetic and biological effects of SAMe supplementation and to explore potential mechanisms underlying the observed biomarker trends.

## 5 CONCLUSION

Oral SAMe at 400 mg daily was safe and well tolerated in people with MCI or dementia due to AD, but did not produce significant changes in cognitive function or AD‐related plasma biomarkers. Our findings suggest that SAMe monotherapy is unlikely to confer disease‐modifying benefits at this dose and duration. Future studies could explore alternative dosing strategies, longer treatment periods, or more stable formulations, but the current data do not support efficacy of SAMe as a stand‐alone therapy for AD.

## CONFLICT OF INTEREST STATEMENT

N.Y. has received honoraria for educational activities from Eli Lilly and Novo Nordisk. No other authors have conflicts of interest to declare. Author disclosures are available in the Supporting Information.

## CONSENT STATEMENT

All participants provided written informed consent prior to any study‐related procedures.

## Supporting information



Supporting Information

Supporting Information

## Data Availability

The individual participant data that underlie the results reported in this article, after de‐identification, will be made available upon reasonable request.
