# Supplementary material for: A phase 2, randomized, multicenter, double‐blind, placebo‐controlled trial of S‐adenosyl methionine in participants with mild cognitive impairment or dementia due to Alzheimer's disease
Source: Alzheimers Dement. 2026 Apr 14;22(4):e71381. doi: 10.1002/alz.71381 (PMC13079069; doi:10.1002/alz.71381)
Supplement: Supplementary file 1 — Supporting Information [file ALZ-22-e71381-s001.docx]

**SUPPLEMENTARY MATERIAL**

**SUPPLEMENTARY TABLE 1 – Differences in baseline characteristics comparing those who did and did not fall within the principal stratum analysis**

|  | **Principal stratum (*n* = 57)** | **Out of principal stratum (*n* = 6)** |
| --- | --- | --- |
| Age, years (median, IQR) | 77 (72, 80) | 79 (67, 85) |
| Female sex (*n*, %) | 29 (50.88) | 4 (66.67) |
| Years of education (median, IQR) | 15 (12, 15) | 11.5 (10, 16) |
| Participants with ≥1 cardiovascular disease/risk factor* (*n*, %) | 42 (73.68) | 5 (83.33) |
| **AD diagnosis (MCI vs dementia) (*n*, %)** |  | |
| AD dementia | 30 (52.63) | 4 (66.67) |
| MCI | 27 (47.37) | 2 (33.33) |
| Use of AD medications† (*n*, %) | 25 (43.86) | 3 (50) |
| MMSE (median, IQR) | 24 (22, 27) | 23 (21, 28) |
| MoCA (median, IQR) | 19 (17, 22) | 21 (11, 25) |
| RBANS (mean, SD) | 68.77 (14.02) | 69.5 (22.22) |
| GDS-15 (median, IQR) | 2 (1, 3) | 1 (1, 3) |
| CDR global (median, IQR) | 0.5 (0.5, 1), n=56 | 0.75 (0.5, 1) |
| Baseline ptau217 (mean, SD) | 0.75 (0.45) | 0.79 (0.45) |
| ***APOE* e4 status** |  |  |
| Heterozygous (n, %) | 37 (64.91) | 3 (50) |
| Homozygous (n, %) | 5 (8.77) | 0 (0) |
| Non-*APOE* e4 carriers (n, %) | 15 (26.31) | 3 (50) |

*Any of hypertension, dyslipidaemia, diabetes, ischaemic heart disease, peripheral vascular disease, stroke, transient ischaemic attack, atrial fibrillation.

†Cholinesterase inhibiters or memantine

AD, Alzheimer’s disease; CDR, Clinical Dementia Rating; GDS, Geriatric Depression Scale; IQR, interquartile range; MMSE, Mini-Mental State Examination; MoCA, Montreal Cognitive Assessment; RBANS, Repeatable Battery for the Assessment of Neuropsychological Status; SD, standard deviation.

**SUPPLEMENTARY TABLE 2 – Baseline characteristics of all randomized participants.**

|  | **SAMe (*n* = 32)** | **Placebo (*n* = 31)** |
| --- | --- | --- |
| Age, years (median, IQR) | 76.5 (71.0, 81.5) | 77 (72, 80) |
| Female sex (*n*, %) | 17 (53.12) | 16 (51.61) |
| Years of education (median, IQR) | 15 (11, 15.5) | 15 (11, 16) |
| Participants with ≥1 cardiovascular disease/risk factor * (*n*, %) | 24 (75) | 23 (74.19) |
| **AD diagnosis (MCI vs dementia) (*n*, %)** |  | |
| AD dementia | 16 (50 ) | 18 (58.06) |
| MCI | 16 (50) | 13 (41.94) |
| Use of AD medications† (*n*, %) | 14 (43.75) | 14 (45.16) |
| MMSE (median, IQR) | 24 (23, 25) | 24 (22, 28) |
| MoCA (median, IQR) | 19 (17, 22) | 20 (17, 23) |
| RBANS (mean, SD) | 65.97 (12.08) | 71.81 (16.75) |
| GDS-15 (median, IQR) | 2 (1, 3.5) | 2 (1, 3) |
| CDR global (median, IQR) | 0.5 (0.5, 1) | 0.5 (0.5, 1) |
| Baseline ptau217 (mean, SD) | 0.78 (0.54) | 0.72 (0.34) |
| ***APOE* e4 status** |  |  |
| Heterozygous (*n*, %) | 22 (68.75) | 18 (60) |
| Homozygous (*n*, %) | 2 (6.25) | 3 (10) |
| Non-*APOE* e4 carriers (*n*, %) | 8 (25) | 10 (32.26) |

*Any of hypertension, dyslipidaemia, diabetes, ischaemic heart disease, peripheral vascular disease, stroke, transient ischaemic attack, atrial fibrillation.

†Cholinesterase inhibiters or memantine

AD, Alzheimer’s disease; CDR, Clinical Dementia Rating; GDS, Geriatric Depression Scale; IQR, interquartile range; MMSE, Mini-Mental State Examination; MoCA, Montreal Cognitive Assessment; RBANS, Repeatable Battery for the Assessment of Neuropsychological Status; SD, standard deviation.

**SUPPLEMENTARY TABLE 3: Fold-change in plasma p-tau217*:**

| **Group** | **n** | **Mean fold change (SD)** | **Median fold change (IQR)** | **Range** |
| --- | --- | --- | --- | --- |
| Placebo | 26 | 1.25 (0.95) | 1.05 (0.87, 1.15) | 0.08 – 5.26 |
| SAMe | 31 | 1.53 (1.59) | 1.05 (0.81, 1.38) | 0.31 – 8.92 |

*Adjusting for baseline, SAMe was associated with a non-significant mean increase of 0.38 in fold-change compared with placebo (95% CI -0.33, 1.08; p = 0.29).

IQR, interquartile range; SD, standard deviation

**SUPPLEMENTARY TABLE 4: Mean percentage change in p-tau217 concentration between SAMe and placebo within each formulation subgroup*:**

| **Formulation received** | **n (SAMe, placebo)** | **Difference in mean % change of ptau217 over 180 days, SAMe vs placebo (95% CI)** | **P-value** |
| --- | --- | --- | --- |
| **Formulation 1** (over-encapsulated tablets) | 27 (14, 13) | 11.6 (-52.9, 76.2) | 0.71 |
| **Formulation 2** (liquid soft gels) | 23 (12, 11) | 75.7 (-97.5, 249.0) | 0.37 |
| **Combination** (formulation 1, then formulation 2) | 7 (5, 2) | 32.6 (-73.0, 138.1) | 0.44 |

*There was no statistically significant interaction between treatment and formulation type (F(3,50) = 0.27, p = 0.844).

**SUPPLEMENTARY TABLE 5 – Description of serious adverse events**

| **Patient ID number** | **SAE description** | **SAE criteria** | **Severity** | **Relation to study drug** | **Action taken with study drug** | **Outcome** |
| --- | --- | --- | --- | --- | --- | --- |
| 1-027 | Transient ischaemic attack | Requires or Prolongs Hospitalisation | Mild | Definitely not related | None | Recovered/Resolved |
| 2-020 | Myocardial ischaemia | Requires or Prolongs Hospitalisation | Mild | Definitely not related | None | Recovered/Resolved |
| 2-035 | Ascending cholangitis | Requires or Prolongs Hospitalisation | Severe | Definitely not related | Interrupted | Recovered/Resolved |

SAE, serious adverse event.

**SUPPLEMENTARY FIGURE 1 - Box plots summarising per cent change in p-tau217 from baseline to 180 days for both intervention and placebo groups for all randomized participants who provided endpoint blood samples (regardless of drug adherence; *n* = 60)**


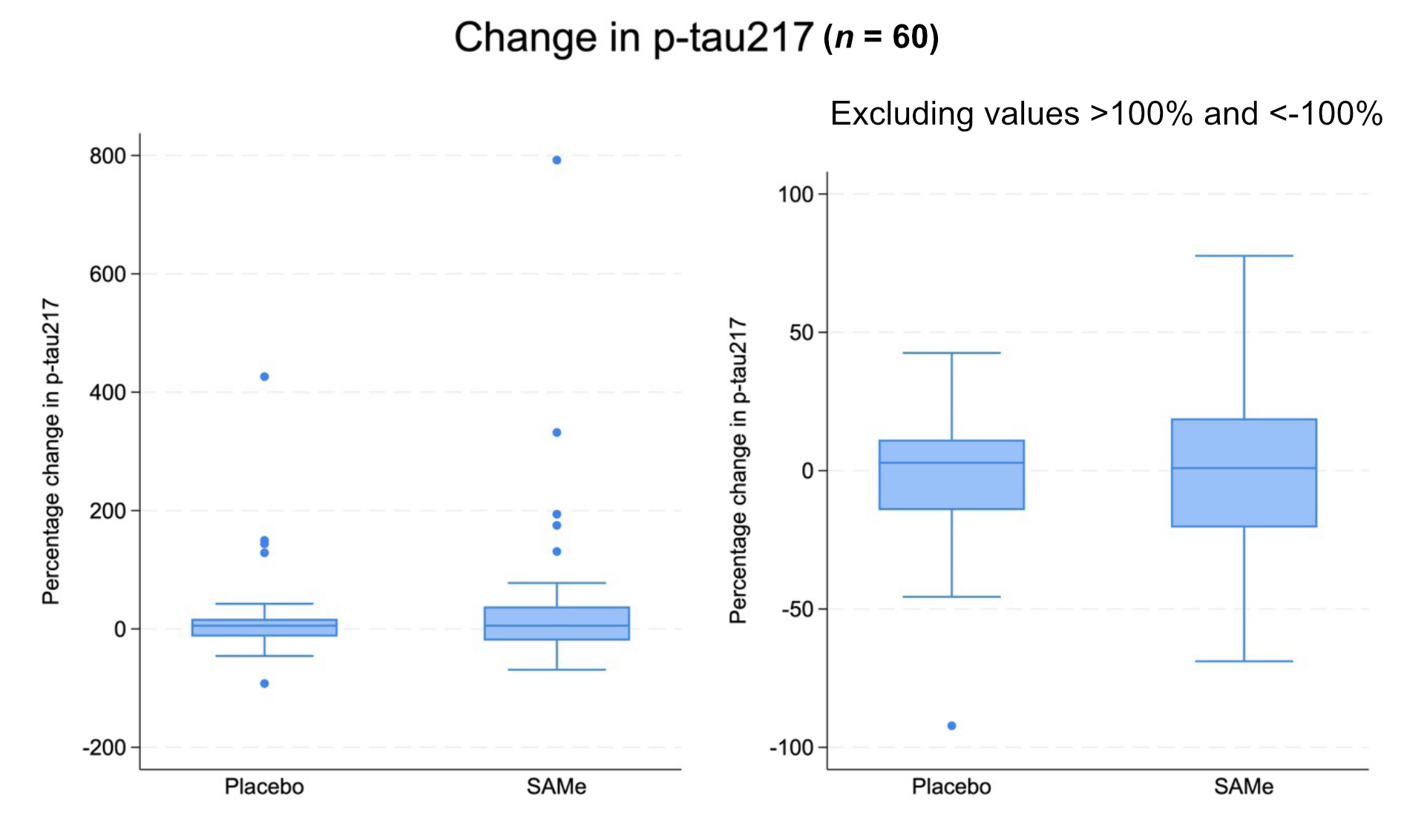


Box plots show the median (line within box), interquartile range (IQR; box upper and lower margins), whiskers (range within 1.5×IQR), and outliers (dots). Negative per cent means reduction in p-tau217 concentration.

SAMe, S-adenosyl methionine.

**SUPPLEMENTARY FIGURE 2 - Box plots displaying changes in secondary endpoints (i.e. RBANS total scaled score, p-tau181, GFAP, NfL) for all randomized participants who provided endpoint blood samples (regardless of drug adherence)**

**
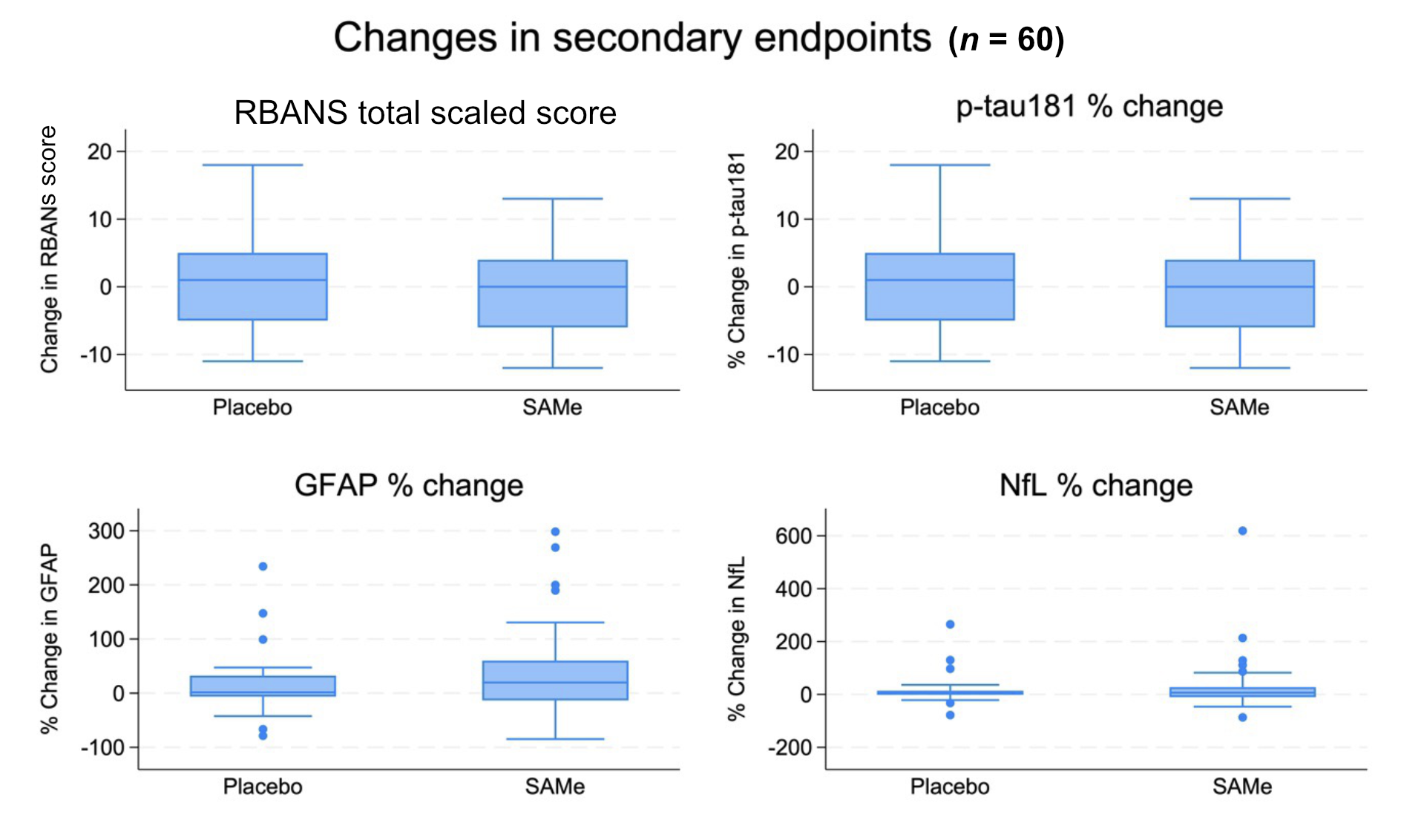
**

Box plots show the median (line within box), interquartile range (IQR; box upper and lower margins), whiskers (range within 1.5×IQR), and outliers (dots). Negative per cent means reduction in biomarker concentration.

GFAP, glial fibrillary acidic protein; NfL, neurofilament light chain; RBANS, Repeatable Battery for the Assessment of Neuropsychological Status; SAMe, S-adenosyl methionine.
